# Ultrasonic Heating Detects Lipiodol Deposition within Liver Tumors after Transarterial Embolization: An In Vivo Approach

**DOI:** 10.3390/biology10090901

**Published:** 2021-09-12

**Authors:** Natsuhiko Saito, Toshihiro Tanaka, Kiyoyuki Minamiguchi, Ryosuke Taiji, Hideyuki Nishiofuku, Takeshi Matsumoto, Toshiko Hirai, Kimihiko Kichikawa, Naoki Kawahara, Daiki Matsuda, Iwaki Akiyama

**Affiliations:** 1 Department of Radiology and Nuclear Medicine, Nara Medical University, Kashihara Nara 634-8521, Japan; totanaka@naramed-u.ac.jp (T.T.); kiyo829@naramed-u.ac.jp (K.M.); rtaiji1119@gmail.com (R.T.); hmn@naramed-u.ac.jp (H.N.); t.matsumoto@naramed-u.ac.jp (T.M.); thirai@naramed-u.ac.jp (T.H.); kkichika@naramed-u.ac.jp (K.K.); 2 Department of Medical Ultrasound Research Center, Doshisha University, Kyotanabe Kyoto 610-0321, Japan; ctuf1012@mail4.doshisha.ac.jp (N.K.); daiorun077@gmail.com (D.M.); iakiyama@mail.doshisha.ac.jp (I.A.)

**Keywords:** ultrasonic heating, temperature coefficient of the sound velocity, TACE, TAE, Lipiodol, liver tumor

## Abstract

**Simple Summary:**

The accumulation of Lipiodol (ethiodized oil) after transarterial embolization is known to reflect tumor necrosis. In general, the treatment effect is evaluated by computed tomography; there has been no development in imaging modalities for several decades. A new technique, ultrasonic heating, can differentiate biological tissues based on the fact that tissues’ characteristic sound velocity varies depending on the temperature. This technique could have the potential to evaluate treatment effect after transarterial embolization as an alternative to computed tomography.

**Abstract:**

Computed tomography (CT) is the standard method to evaluate Lipiodol deposition after transarterial embolization (TAE) for a long period. However, iodine but not Lipiodol can be observed on CT. A minimally invasive other method to detect Lipiodol has been needed to evaluate accurate evaluation after procedure. The purpose of this study was to evaluate the efficacy of using the rate of change in sound velocity caused by ultrasonic heating to reflect Lipiodol accumulation after TAE in a rat liver tumor model. We analyzed the association of this developed technique with CT images and histological findings. Eight rats bearing N1S1 cells were prepared. After confirmation of tumor development in a rat liver, Lipiodol was injected via the hepatic artery. Seven days after TAE, CT scan and sound velocity changes caused by ultrasonic heating were measured, and then the rats were sacrificed. An ultrasonic pulse-echo method was used to measure the sound velocity. The temperature coefficient of the sound velocity in each treated tumor was evaluated and compared with the mean CT value and the histological Lipiodol accumulation ratio. Pearson’s correlation coefficients were calculated to assess the correlation between the measured values. The correlation coefficient (r) of the mean CT value and histological Lipiodol accumulation ratio was 0.835 (*p* = 0.010), which was considered statistically significant. Also, those of the temperature coefficient of the sound velocity and the histological Lipiodol accumulation ratio were statistically significant (r = 0.804; *p* = 0.016). To our knowledge, this is the first study that reported the efficacy of ultrasonic heating to detect Lipiodol accumulation in rat liver tumors after TAE. Our results suggest that the rate of change in sound velocity caused by ultrasonic heating can be used to evaluate Lipiodol accumulation in liver tumors after TAE, and thus could represent an alternative to CT in this application. This new innovative technique is easy to treat and less invasive in terms of avoiding radiation compared with CT.

## 1. Introduction

Liver cancers are the fourth most common cause of cancer-related death and rank sixth in terms of incident cases in the world [1]. Most primary liver cancer is hepatocellular carcinoma (HCC). HCC-related mortality continues to increase despite the overall declining trends in cancer incidence and death rates [2]. Transarterial chemoembolization (TACE) was shown to improve overall survival in patients with nonresectable HCC in randomized controlled studies performed in Europe and Asia [3]. In the real world, TACE is widely performed.

Conventional TACE involves the injection of an emulsion of an anticancer drug solution with ethiodized oil (Lipiodol Ultra-Fluide; Guerbet, Villepinte, France) followed by gelatin particle embolization, and is widely performed for the treatment of unresectable hepatocellular carcinoma (HCC) [4,5,6,7,8]. It is known that the Lipiodol accumulation in a tumor reflects the tumor necrosis in the surgical specimen after TACE [9]. The treatment effect of TACE is usually evaluated by computed tomography (CT), which has been a standard imaging modality for more than 25 years [10]. There are many published studies regarding the correlation between CT images after TACE and therapeutic outcome [9,11,12,13,14,15,16,17,18,19,20]. However, most of them analyze the pattern of Lipiodol deposition or CT values in the targeted tumors. This may not directly represent the volume or density of oil in the tumor and could just represent the iodine concentration. To date, no other imaging modalities have been developed for the evaluation of Lipiodol deposition after TACE.

Recently, ultrasonic heating has seen some development as a modality. The fact that a tissue’s characteristic sound velocity varies depending on the temperature can be used to obtain information about the tissue [21]. When biological tissue is exposed to ultrasound, the temperature rises depending on physical parameters inherent to the tissue (e.g., specific heat, thermal diffusion coefficient, attenuation coefficient, ultrasonic intensity). Tissue characterization based on the rate of temperature increase due to ultrasonic heating has been proposed [22]. This method has the potential to facilitate the in vivo evaluation of the volumetric distribution of Lipiodol inside tumors, since Lipiodol primarily consists of poppy-seed oil. A previous in vitro study demonstrated that tumors implanted in a rat liver and Lipiodol had different temperature coefficients of the sound velocity [23].

Tsujimoto et al. measured the sound velocity of rat liver, and showed that there was a positive temperature coefficient near body temperature. They reported that Lipiodol’s temperature coefficient of the sound velocity showed a negative temperature coefficient [23]. On the other hand, the amount of heat generated by locally heating biological tissue by exposure to ultrasonic waves is proportional to the product of the ultrasonic attenuation coefficient and the ultrasonic intensity, and the temperature change caused by ultrasonic exposure is the heat production rate. It depends on diffusivity, time constant for perfusion, and heat capacity per unit volume. This relationship is known as the biothermal transport equation [24]. The rate of change in tissues’ sound velocity due to short-term ultrasonic exposure can be estimated by the ultrasonic pulse-echo method [25]. Tsujimoto et al. estimated the rate of change in sound velocity by transmitting ultrasonic pulses before and after heating animal tissues and comparing the phases of the echoes [26]. By measuring the rate of change in sound velocity before and after ultrasonic heating in this way, it is possible not only to characterize whether the tissue is adipose or non-adipose tissue, but also measure the degree of Lipiodol infiltration of tumors that are non-adipose tissue infiltrated with Lipiodol due to the difference of temperature coefficient of the sound velocity. 

The purpose of this pilot in vivo study was to evaluate the association between the temperature coefficients of the sound velocity obtained by ultrasonic heating and the histological Lipiodol accumulation in tumors after Lipiodol injection, and to compare the accuracy of this developed technique with CT image reflecting Lipiodol volume. For simplicity, the procedure in this study only used an injection of Lipiodol without an anticancer drug, and therefore, the procedure was generally named transarterial embolization (TAE) not TACE. In the following description, the name of this procedure is unified with TAE.

## 2. Materials and Methods

### 2.1. Cell Line

The N1-S1 (CRL-1604; ATCC, Manassas, VA, USA) rat tumor cell lines were obtained and cultured in Iscove’s modified Dulbecco’s medium supplemented with 10% fetal bovine serum and glutamic acid (RPMI-1640 with L-glutamine, phenol red, and HEPES). After about five or six passage, N1-S1 cells were injected to rat liver.

### 2.2. Animal Model and Study Design

This study was approved by the Animal Experimentation Committee of our institution. In addition, all experiments were performed in accordance with Animal Care Guidelines of our institution.

We used eight male Sprague Dawley rats, 7 to 9 weeks old, weighing approximately 300–400 g (purchased from CLEA Japan, Inc., Tokyo, Japan). All procedures were performed while rats were well-anesthetized with isoflurane (induced in 4% and maintained in 2%) through their nose. The N1S1 cells were implanted in the surface of left lobe of the liver under laparotomy. Implantation was performed by making local wheal on the surface of liver parenchyma. An amount of 4 × 10^7^ cells was prepared in 200 µL medium and the whole quantity was implanted. Immediately after injection, compression using cotton swab for a few minutes was performed. Then, the needle hole was cauterized for a moment with handheld electrocautery (Bovie Medical, Clearwater, FL, USA) to prevent tumor cell reflux or bleeding. After checking hemostasis, the skin wound was sutured with 2–0 nylon sutures. One week after implantation, a small laparotomy was performed and the development of tumor was visually checked and the tumor size was measured. Then, Lipiodol-TAE was performed. One week after TAE, a CT (Cosmoscan FX, Rigaku, Tokyo, Japan) scan was obtained and the temperature coefficient of the sound velocity was measured by ultrasonic heating. Following this, rats were sacrificed for histological analysis.

### 2.3. TAE Procedure

Inhalation anesthesia was used with isoflurane during the procedure. A skin cut of 1 to 2 cm was performed in the left paramedian region of the neck. Under the skin, muscles were dissected. Then, a microscope (SZX7; Olympus, Tokyo, Japan) was introduced. Under the microscope, the left common carotid artery was exposed by exfoliating the surrounding connective tissues. The distal side of left common carotid artery was ligated with a 2–0 silk suture (Alfresa Pharma, Osaka, Japan). In the proximal side, a loop surrounding the vessel was made to prevent bleeding when puncture failed. A 20-gauge Angiocath (BD Vascular Access, Franklin Lakes, NJ, USA) was used for puncture. During puncture, a silk suture of distal side was pulled to make tension for common carotid artery. After successful cannulation, a custom-made 1.6/1.8 Fr 40 cm-long microcatheter (Tokai Medical Products, Kasugai, Japan) and a 0.014 inch guidewire (BEGIN PLUS; Asahi Intecc, Seto, Japan) were inserted into the descending aorta under fluoroscopy. Then, the celiac artery was selected with a guidewire which was bended as a J-shape to make it easier to hook. Through the guidewire, microcatheter was inserted into left hepatic artery [27]. Through the catheter, digital subtraction angiography (DSA) was performed using iopamidol (Iopamirom 300; Bayer, Osaka, Japan) to visualize the tumor stain and disclose vessel anatomy. Then, Lipiodol-TAE was performed. Lipiodol was injected using a 1 cc syringe until stasis of blood flow.

### 2.4. CT Scan and Image Evaluation

The scanning parameters were as follows: 90 kVp, 88 µA; scan speed, 18 s to 2 min; minimum slice thickness: 10 µm; view rate, 28 frames/second. After scanning, images were reconstructed with a 72 × 72 × 72 mm^3^ field of view (matrix size 512 × 512 × 512) and a pixel size of 144 µm. The method for evaluating the ratio of Lipiodol accumulation on CT was as described by Haubold et al. [28]. Hounsfield units (HUs) were measured by plotting the region of interest (ROI) around the iodine deposition of target lesions. A total of 6 points with equal slice interval were selected to measure the ROI. After that, the mean density of every slice was multiplied by the area of the iodine deposition and they were summed to quantify the total density through the volume of the iodine deposition. Afterwards, the density through the volume was evaluated to analyze the relative density of the iodine deposition. All of the above measurements were performed using Synapse Vincent (Fujifilm, Tokyo, Japan).

### 2.5. Method for the Measurement of the Rate of Change in Sound Velocity Caused by Ultrasonic Heating and the Statistical Analysis of Acquired Data

**Figure 1:**
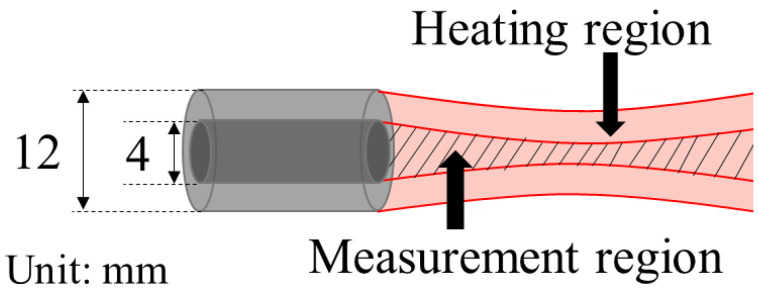
Schematic illustration of the integrated probe of coaxial transducers for heating and measurement.

Based on Tsujimoto’s report on the rate of change in sound velocity of biological tissue after exposure to ultrasonic waves for 100 ms at 3.2 MHz in a focal region [26], the experimental system in this study was constructed as follows. We prototyped a confocal concentric two-split integrated ultrasonic probe in which the focal region of the ultrasonic beam for heating and the focal region of the ultrasonic beam for measurement coincided with each other [26]. Figure 1 shows the schematic illustration of two beams formed by the integrated probe, and Table S1 shows the specifications of the transducers. 

The rate of change in the sound velocity in biological soft tissue is about 0.1% when the temperature is increased by 1 °C due to ultrasonic heating. The ratio of the echo delay time difference before and after heating to the ultrasonic wave propagation time is also about 0.1% [26]. Since the thickness of the tissue specimens was about 5 mm, the reciprocating propagation time of the ultrasonic waves would be 6.6 μs at a speed of sound of 1500 m/s. Therefore, the delay time difference was estimated as 6.6 ns. In order to measure this time difference with high accuracy, the ultrasonic frequency was set to 10 MHz (period: 100 ns).

It is difficult to measure the time difference of echoes during the heating period, therfore the acquisition of ultrasonic echoes for measurement was carried out before and after the heating period. Additionally, the frequency of the ultrasonic waves used for heating was 5.0 MHz, which was lower than the frequency of the ultrasonic waves used for measurement.

**Figure 2:**
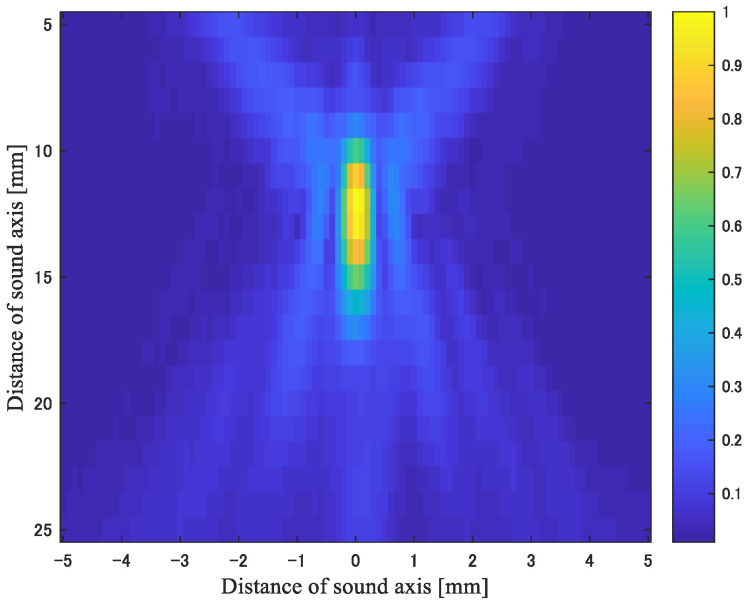
Sound pressure distribution formed by the transducer for heating.

Figure 2 shows the sound pressure field of the heating ultrasonic waves. From Figure 2, it can be seen that the beam width in the focal region was 1 mm in the lateral direction and 5 mm in the range direction.

**Figure 3:**
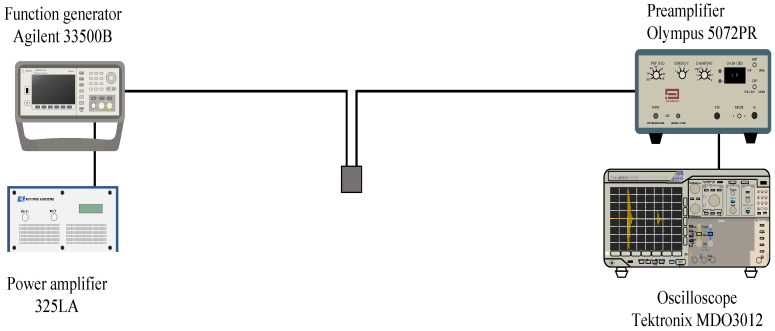
Experimental setup for ultrasonic heating and ultrasonic measurement system.

Because the strongest point of pulse echo is about 12 mm in width, we set the distance from the probe tip to center of the liver tumor to about 12 mm by using B-mode ultrasonography. A confocal concentric two-split integrated ultrasonic probe tip was directly attached to liver parenchyma under laparotomy through ultrasonography jelly. Figure 3 shows the experimental system for this method.

It consists of a transmission system that drives a heating transducer and an ultrasonic pulse transmission/reception system for measuring the rate of change in sound velocity before and after heating by the pulse-echo method. The specifications of each transducer are shown in Table S1. The ultrasonic heating transmission system consisted of an arbitrary waveform generator (Keysight, 33510B) and a power amplifier (E&I, 325LA). The sinusoidal waves generated by the arbitrary waveform generator were amplified by the power amplifier. They drove the transducer for heating. In the measurement signal transmission/reception system, the repetitive and periodic pulsed waves were generated by the pulser (Olympus, 7072PR), the received signal of the echo from the sample was amplified with the preamplifier (Olympus, 7072PR), and it was then received at the oscilloscope (Tektronix, MDO3000). The analog-to-digital converted signal with a sampling frequency of 250 MHz and quantization of 8 bits was transferred to a personal computer (HP Elitebook) connected via USB. The rate of change in sound velocity was calculated by numerical processing on a personal computer. The positive and negative sound pressures of the ultrasonic waves for heating were, respectively, 0.60 and −0.51 MPa, and the exposure time was 10 ms. The sound pressures were measured by a hydrophone (Onda, HNR0500). The ultrasonic intensity *I*_SPPA_ (I: Intensity, SPPA: Spatial Peak Pulse Average) was 10 W/cm^2^. The pulse repetition time of the ultrasonic waves transmitted to measure the rate of change in sound velocity was 1.0 ms. Since the heating ultrasonic waves were transmitted 100 times at 2 s intervals, the *I*_SPTA_ (I: Intensity, SPTA: Spatial Peak Temporal Average) was not very high, as the measured value was 51 mW/cm^2^. Tables S2 and S3 show the parameters for the transmission of ultrasonic waves for heating and pulse-echo reception.

**Figure 4:**
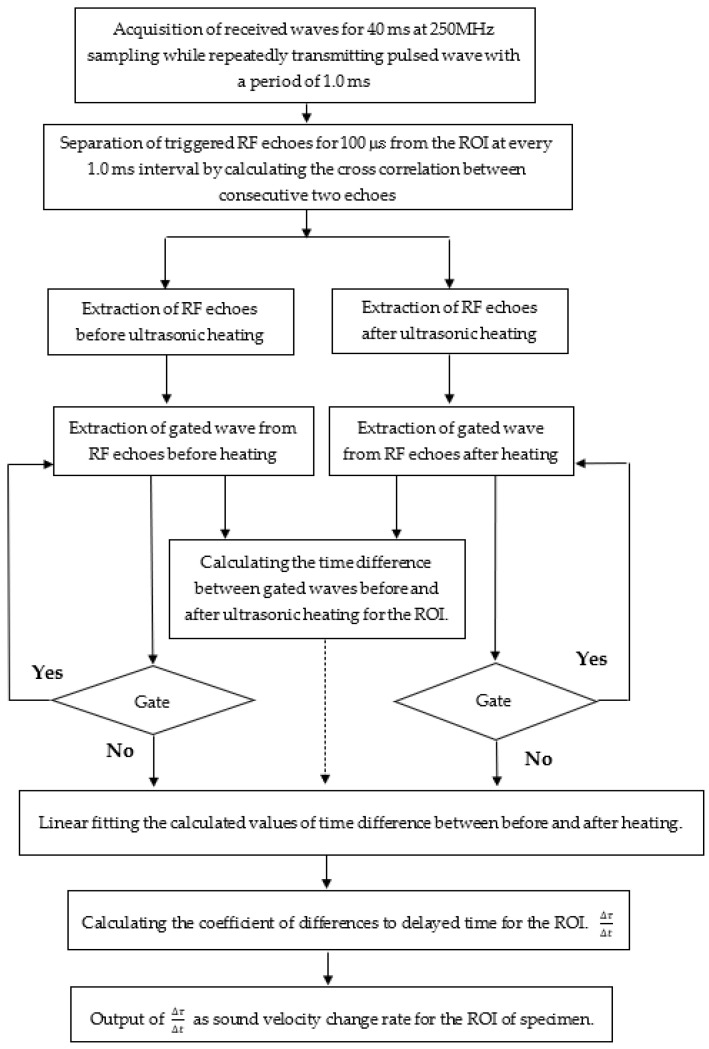
Process flow for the calculation of the rate of change in the sound velocity in the specimen.

The following describes the process for calculating the rate of change in sound velocity. For the two echo signals before and after heating, the signal was extracted by applying a 625 ns rectangular window function (corresponding to 0.5 mm) to the region of the delay time corresponding to the focal region (11 to 13 mm), and the window function was shifted at an interval of 313 ns. After shifting 20 times, the time difference Δτ of the echo before and after heating with respect to the shift Δt of the window function was obtained. The slope Δτ/Δt of the time shift before and after heating with respect to the shift of the window function was equal to the rate of sound velocity change in the focal region. The calculation process is shown in Figure 4. Table S4 shows the numerical values of the parameters set for the calculation.

### 2.6. Histological Analysis

Rats were sacrificed immediately after the completion of experimental measurements. The removed liver tumor and surrounding liver parenchyma were treated with 10% formalin fixation. Two slices close to the center of the tumor were made in each rat. Then, specimens were carried to a laboratory (Nara-byouri Laboratory Co., Ltd., Nara, Japan) to perform staining and make prepared slides. Samples were stained with hematoxylin–eosin (HE) and additional silver nitrate (AgNO_3_) as described in [29]. Then, the slides were scanned with a fluorescence microscope (BZ-X700; Keyence, Osaka, Japan) with a *40 resolution using a *4 objective lens and 8.33 ms exposure. The region of Lipiodol accumulation was delineated by a pure white foam including a black line inside the edge. The histological Lipiodol accumulation ratio was defined as the ratio of this delineated accumulation area to the entire tumor area as calculated by the analysis software (BZ-H3A; Keyence, Osaka, Japan). The average value of the two slices was adopted. Masada et al. evaluated tumor necrosis after TACE by using this software [30].

### 2.7. Statistical Analysis

Statistical analysis was performed using SPSS statistical software, version 26.0 (SPSS, Inc.; Chicago, IL, USA). Pearson’s correlation coefficient was calculated between 2 of the following 3 values: the temperature coefficient of the sound velocity, the CT value, and histological Lipiodol accumulation ratio. *p*-Values less than 0.05 were considered statistically significant.

## 3. Results

**Figure 5:**
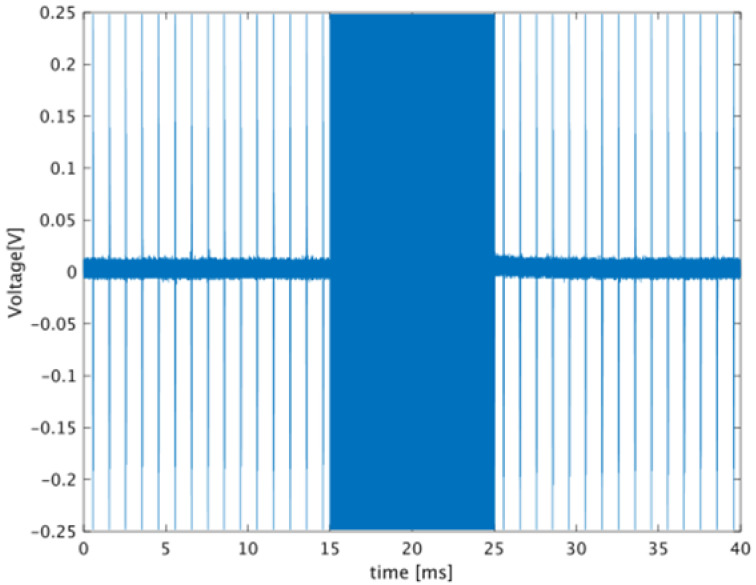
Example of a received waveform from a specimen.

**Figure 6:**
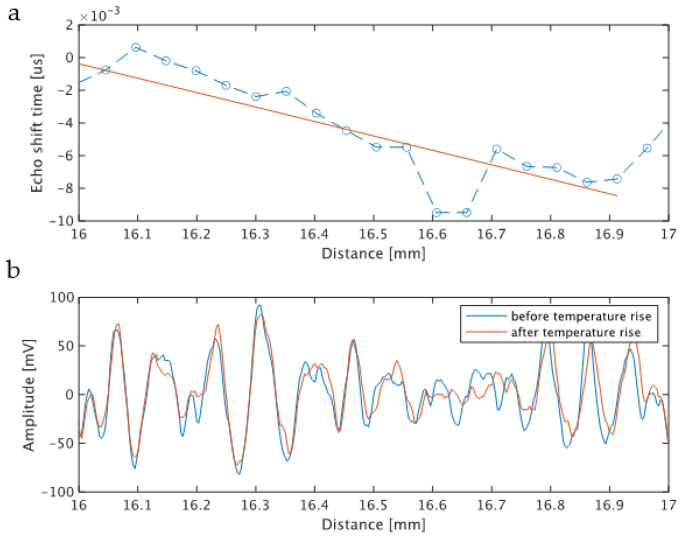
(**a**) Example of the time difference of echoes between before and after heating; (**b**) example of echoes before and after heating from the ROI.

Figure 5 shows an example of the signal received by the transducer. The high-amplitude signal shown in Figure 5 from 15 to 25 ms is the ultrasonic echo for heating. The signals before and after heating are the echoes from 13 and 26 ms in Figure 6, respectively. Next, the waveform corresponding to the echo from the set ROI was extracted by the gate, and the time difference between before and after heating was calculated from the two waveforms extracted at the same time. The time difference was estimated from the phase calculated by orthogonal detection, and the details are shown in [26]. The time difference obtained by the gate shift time on the horizontal axis is plotted on the vertical axis. The linear coefficient calculated by the linear approximation of this plot corresponds to the rate of change in sound velocity. The extracted waveforms before and after heating are shown in Figure 6b.

Tissue motion would affect the values of the sound velocity change measured by the proposed method. To avoid tissue motion error, the exposure time to ultrasonic waves for heating was restricted to 10 ms. Generally, the amplitude of echoes from biological tissue shows stochastic fluctuation. When the amplitude after quadrature detection reaches zero, the error of the phase calculation increases. To avoid errors due to tissue motion and phase calculation, the coefficient data with a significance level of 5% or less were averaged.

In all eight rats, liver tumors were visually detected 1 week after implantation, and all transarterial catheter procedures were successfully performed. All rats were alive 1 week after procedures, and CT scan and ultrasonic heating were conducted to completion. 

The median tumor diameter was 11 mm (5 to 17 mm). The mean value of the change rate of the sound velocity was −0.0017 ± 0.0030 m/s. The mean CT value was 1090.099 ± 391.548 HU. The mean histological accumulation ratio of Lipiodol was 0.136 ± 0.052. Table 1 shows the data. 

**Figure 7:**
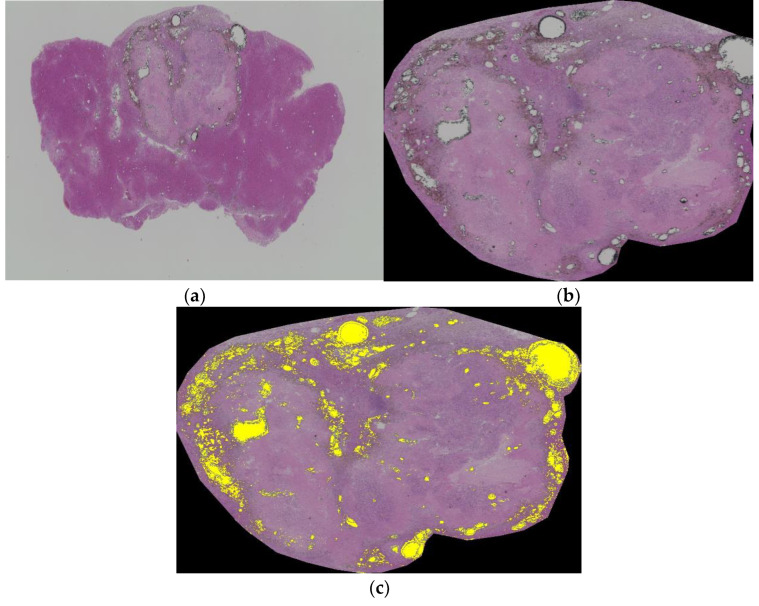
(**a**) One of the slices close to the center of the tumor; (**b**) extracted tumor lesion from (**a**); (**c**) Lipiodol accumulation ratio calculated by the analysis software.

The correlation coefficient (r) of the mean CT value and histological Lipiodol accumulation ratio was 0.835 (*p* = 0.010). The correlation between the temperature coefficient of the sound velocity and histological Lipiodol accumulation ratio was r = 0.804 (*p* = 0.016). The temperature coefficient of the sound velocity was also correlated with the mean CT value (r = 0.893; *p* = 0.003). The representative case is shown in Figure 7a–c. Figure 7a shows one of the slices close to the center of the tumor. Figure 7b is extracted tumor lesion from Figure 7a. Then, the Lipiodol accumulation ratio is calculated by the analysis software (Figure 7c).

## 4. Discussion

To evaluate the tumor response after TACE, CT image has been the most commonly used method. Matsui et al. reported that a CT value in a tumor of less than 270.2 HU was significantly associated with tumor recurrence 1 week after TACE [11]. Recently, a study analyzing the amount of iodine using dual-energy CT was reported. Choi et al. calculated the iodine densities immediately following conventional TACE using spectral CT and proposed a threshold value of 10.68 mg/mL for predicting the tumor responses from the 12-month follow-up images [12]. There are many published studies regarding the correlation between CT images after TACE and therapeutic outcome, however these studies did not directly evaluate the concentration or amount of oil, but rather the concentration of iodine contained in Lipiodol [9,11,12,13,14,15,16,17,18,19,20]. To the best of our knowledge, there is currently no non-invasive imaging technique that can be used to directly evaluate the volume or density of oil in tumors. 

In the previous study, the temperature coefficient of the sound velocity of Lipiodol showed negative values and that of rat liver tumor showed positive values [23]. Therefore, that of rat liver tumor injected with Lipiodol can show various values from negative to positive values depending on the degree of Lipiodol, which can reflect the amount of Lipiodol filled in the liver tumor. In this study, the temperature coefficient of the sound velocity obtained by ultrasonic heating showed a correlation with histologically determined Lipiodol density to some extent. The correlation coefficient of this developed technique seemed to be almost the same as that for the CT value. This result means the change rate of sound velocity caused by ultrasonic heating could be used to evaluate the volume of Lipiodol in tumor tissue and may have the potential to predict tumor necrosis after TAE. Compared with CT, ultrasonography has the advantages that it can be performed more easily and does not use radiation [31].

The TAE procedure in this study was quite similar to that in clinical practice. A microcatheter was selectively inserted into the hepatic artery under fluoroscopic guidance and Lipiodol was manually injected into the tumor-feeding artery until hemostasis. In the clinical context, the mixture of lipiodol and anticancer drug is injected into a tumor-feeding artery through a catheter and then gelatin particles are also injected finally. The purpose of this study was to evaluate the Lipiodol deposition in rat liver tumors, therefore anticancer drug and gelatin particles were not used to simplify the experiment. Because of the absence of an anticancer drug, the procedure in this study was named not transarterial chemoembolization (TACE) but transarterial embolization (TAE). In the future, the efficacy of this technique should be validated in the case of TACE.

This new innovative technique can make frequent examinations after TACE shorter in examination time and less invasive than CT. Patients suffering from liver cancers can be released from the stress of CT. Moreover, because direct Lipiodol deposition is determined by this technique, more precise judgement of treatment efficacy can be achieved.

As for the technique of ultrasonic heating, the MI value calculated from the sound pressure of the ultrasonic waves used for heating was 0.16, the *I*_SPTA_ was 50 mW/cm^2^, and the measured rate of change of the sound velocity was about 0.1%. Since this method uses an approximation assuming that the rate of change in sound velocity is small, we believe that the values used for the heating ultrasonic wave are appropriate. According to international regulation, the MI upper limit is 1.9, and the acoustic intensity upper limit of I_SPTA_ is 720 mW/cm^2^ [32]. Thus, the values in this study are within the permissible output ranges. 

From the viewpoint of ultrasonic beam configuration, it is difficult to design an array probe such that the frequencies of the ultrasonic waves for heating and for measurement are different. Thus, it is important to use the same frequency for both ultrasonic waves. In this study, an ultrasonic beam formed by transducers with different resonance frequencies was used as a confocal probe. However, we demonstrated that phase detection could transmit ultrasonic pulsed waves and receive echoes before and after the ultrasonic exposure for heating. The results of this research indicate that it is not always necessary to separate the ultrasonic frequencies, and it is possible to transmit ultrasonic waves for heating from the same transducer that transmits pulsed waves for measurement. Further study is required to improve the array transducer and signal input as well as the output hardware design.

This study had several limitations. First, only a small number of samples were analyzed for the pilot study. Second, the ultrasonic heating device was directly exposed to rat liver under laparotomy, therefore the effect of heat injury or ultrasonic attenuation on the skin was not measured. Third, the tumor necrosis ratio was not evaluated due to the short follow-up period. In the future before clinical application, these limitations should be overcome.

## 5. Conclusions

In conclusion, the new technique of ultrasonic heating can be effective to evaluate the therapeutic effect after TAE. Considering the precise predictability of the Lipiodol accumulation in this pilot animal study, this technique can replace CT with revising some points; 1. Use a mixture of Lipiodol and an anticancer drug (TACE), 2. Use the indirect irradiation of echo pulse through the skin and connective tissue, and 3. Confirm the efficacy of detecting Lipiodol on a monthly basis or more.

## Figures and Tables

**Table 1:** The data of the distribution of the mean CT value, the temperature coefficient of the sound velocity, and the histological Lipiodol accumulation.

Number of Rats	The Mean CT Values (HU)	The Temperature Coefficient of the Sound Velocity (10^−3^ m/s)	The Histological Lipiodol Accumulation (%)
No. 1	1830.77	−6.29	18.7
No. 2	685.90	1.74	8.8
No. 3	922.59	−2.74	9.2
No. 4	1283.26	−4.90	22.1
No. 5	805.39	−0.02	10.7
No. 6	1548.20	−4.21	18.9
No. 7	908.44	1.43	13.4
No. 8	736.23	1.44	7.3

HU: Hounsfield Unit.

## Data Availability

The data that support the findings of this study are available from the corresponding author (N.S.) upon reasonable request.

## Supplementary Materials

The following are available online at www.mdpi.com/article/10.3390/biology10090901/s1, Table S1: Specification of the two transducers for heating and measurement, Table S2: Parameters of the transmitted ultrasonic waves used for heating, Table S3: Parameters of the transmitted ultrasonic waves used for measurement, Table S4: Parameters of setup values for calculation of sound velocity change.
