# Supplementary material for: Ultrasonic Heating Detects Lipiodol Deposition within Liver Tumors after Transarterial Embolization: An In Vivo Approach
Source: Biology (Basel). 2021 Sep 12;10(9):901. doi: 10.3390/biology10090901 (PMC8466351; doi:10.3390/biology10090901)
Supplement: Supplementary file 1 [file biology-10-00901-s001.zip › biology-1331264-SI.pdf]

Table S1. Specification of the two transducers for heating and measurement.

Usage	Resonant Frequency [MHz]	Diameter [mm]	Focal Distance [mm]
Heating	5.0	12.0	12.0
Measurement	10.0	4.0	12.0

Table S2. Parameters of the transmitted ultrasonic waves used for heating.

	**Frequency [MHz]**	**Positive and Negative Sound Pressure [MPa]**	**I_{SPTA} [mW/cm^2]**	**Wave Duration [ms]**	**Interval of Exposure [s]**	**Number of Exposures**
Burst Wave	5.0	0.60, -0.51	51	10.0	2.0	100

I: Intensity, SPPA: Spatial Peak Pulse Average.

Table S3. Parameters of the transmitted ultrasonic waves used for measurement.

**Transmitted Wave Type**	**Center Frequency [MHz]**	**Pulse Duration [ms]**	**Pulse Repetition Time[ms]**
Periodic Pulsed Wave	10	<0.5	1.0

Table S4. Parameters of setup values for calculation of sound velocity change.

**Rectangular Window Size[ns]**	**Window Shift Interval[ns]**	**Frequency of Quadrature Detection [MHz]**	**Number of Window Shifts**
619	313	10	20
